# Surface-Modified Nanofibrous PVDF Membranes for Liquid Separation Technology

**DOI:** 10.3390/ma12172702

**Published:** 2019-08-23

**Authors:** Evren Boyraz, Fatma Yalcinkaya, Jakub Hruza, Jiri Maryska

**Affiliations:** 1 Center for nanomaterials, Advanced Technology and Innovation, Department of Nanomaterials and Informatics, Technical University of Liberec, Studentska 1402/2, 46117, Czech Republic; 2 Faculty of Mechatronics, Institute for New Technologies and Applied Informatics, Technical University of Liberec, Studentska 1402/2, 46117 Liberec, Czech Republic

**Keywords:** PVDF, membrane, surface modification, filtration, nanofiber, electrospinning

## Abstract

Preparing easily scaled up, cost-effective, and recyclable membranes for separation technology is challenging. In the present study, a unique and new type of modified polyvinylidene fluoride (PVDF) nanofibrous membrane was prepared for the separation of oil–water emulsions. Surface modification was done in two steps. In the first step, dehydrofluorination of PVDF membranes was done using an alkaline solution. After the first step, oil removal and permeability of the membranes were dramatically improved. In the second step, TiO_2_ nanoparticles were grafted onto the surface of the membranes. After adding TiO_2_ nanoparticles, membranes exhibited outstanding anti-fouling and self-cleaning performance. The as-prepared membranes can be of great use in new green separation technology and have great potential to deal with the separation of oil–water emulsions in the near future.

## 1. Introduction

Burgeoning industrial development unavoidably generates large volumes of wastewater that contain emulsified oil/water mixtures. Cost-effective and efficient separation processes for such mixtures are in high demand yet still challenging. Current separation techniques include centrifuges, magnetic separation, oil skimming, floating, and depth filters, which are more suitable for immiscible oil/water mixtures but not for emulsified ones [1]. Emulsified oil/water mixtures can contain droplet sizes less than a few microns, which require specific separation techniques.

Polymer-based microfiltration (MF) membranes were successfully used for the separation of oil/water emulsions. However, the permeability and flux of the membrane decline rapidly due to membrane fouling that reduces their performance over a short operation time. The main reason for the membrane fouling is that oil droplets plug the pore size of the membrane and/or adsorption of the surfactant. To address the membrane fouling problem, several attempts were made to improve the hydrophilicity of the membrane through blending of hydrophilic polymers, surface grafting, or surface modification. Zhang et al. [2] prepared an alkaline-induced phase inversion polyacrylonitrile (PAN) membrane, which showed superhydrophilic/underwater superoleophobic characteristics. During the phase inversion process, sodium hydroxide (NaOH) was added to the coagulation bath. In the NaOH coagulation bath, the –CN groups of PAN hydrolyzed to –COOH groups, which introduced hydrophilic components to the PAN. Moreover, adding NaOH led to the formation of a rough structure on the membrane surface. The resultant membrane showed very high flux with oil rejection of the oil residual. On the other hand, Fan et al. [3] prepared hydrophilic/oleophilic polystyrene (PS)/polyacrylonitrile (PAN) bicomponent membranes that exhibited extremely high oil flux. The bicomponent PS/PAN membrane was prepared using the electro-blowing method. Results indicated that the flux of the membrane achieved up to 1800 L∙m^−2^∙h^−1^ (efficiency > 99.6%) with a flux recovery ratio of 94.09% after 10 cycles. Moreover, the tensile strength of the membrane improved by increasing the ratio of PAN in the mixture. In another work [4], titanium dioxide (TiO_2_) nanoparticles were grafted onto the surface of polyvinylidene fluoride (PVDF)/polyacrylonitrile (PAN) membranes using a two-step modification system. In the first step, hydroxyl and carbonyl groups were introduced onto the membrane surface using low-vacuum argon plasma treatment and a NaOH aqueous solution. In the second step, TiO_2_ nanoparticles were grafted onto the surface of the membrane. Results indicated that the TiO_2_ grafted membrane showed extremely high water permeability with a self-cleaning property.

Nanofiber webs are a good candidate for use in filtration applications due to their high surface area, tight pore size, and highly porous structure. Even though there is an air filtration application on an industrial scale, water domain applications are still in progress. Mechanical strength of the nanofibers is not enough to withstand any pressure under water. There were a number of works submitted on improving mechanical properties of nanofibers such as dip coating, addition of epoxy, polymer or inorganics blending, tailoring, ultrasonic welding, heat pressing, etc. [5,6,7,8,9,10,11,12,13,14].

Herein, the mechanical problem of the nanofiber layer was solved using the heat-press lamination process. Using this method, nanofiber webs were transported and adhered to a different surface without any damage [15]. A PVDF nanofibrous membrane was used for the separation of oil/water emulsions. PVDF is commonly used in membrane technology due to its outstanding mechanical, chemical, thermal, and oxidation resistance properties [16]. PVDF is an oleophilic/hydrophobic membrane due to its low surface energy (25 dynes∙cm^−1^) [16,17]. The aim of this work was to prepare self-cleaning PVDF nanofibrous hybrid membranes for the separation of oily wastewater.

For this reason, various nanofiber layers were used for the separation of oil–water emulsions. Firstly, performance of the membranes was measured and compared. Secondly, selected membranes were carried to the surface modification process. Finally, the self-cleaning membrane was prepared for the separation of oil–water emulsions.

## 2. Materials and Methods

### 2.1. Membrane Preparation

PVDF nanofibers were obtained from Nanocenter (Laboratory of nanomaterial application, Technical University of Liberec, Liberec, Czech Republic). Nanofiber layers had densities of 1, 2, and 3 g/m^2^. To increase mechanical strength, a 100-g/m^2^ polyethylene terephthalate nonwoven (Mogul Nonwovens, Gaziantep, Turkey) was used as a supporter. A co-polyester adhesive was used to bind nanofibers to the nonwoven surface. The heat-press method was applied as explained previously [8,13,15]. The highest-density nanofiber web was taken for further surface modification. Sample abbreviations are given in Table 1.

### 2.2. Surface Modification

The first step in the surface modification was done by dehydrofluorination of the PVDF membrane using alkaline solutions. Two types of dehydrofluorinated PVDF were prepared with sodium hydroxide (NaOH, Penta s.r.o., Prague, Czech Republic) and potassium hydroxide (KOH, Penta s.r.o., Prague, Czech Republic).

The reaction of the alkaline solution with PVDF is given as follows [18]:

–(CH_2_–CF_2_)– + xOH → –(CH=CF)– + xF + H_2_O,

where x is Na or K.

After the lamination process, one of the membranes was immersed into 72 g of NaOH solution in 30 mL of distilled water (DI) for 48 h, while the other membrane was immersed into 2 g of KOH solution in 20 mL of isopropyl alcohol (IPA, Penta s.r.o., Prague, Czech Republic) for 1 h.

Subsequently, the membranes were taken from the alkaline solutions, washed several times with DI, and immersed into 0.5 g of titanium dioxide (TiO_2_, 20 nm, Sigma-Aldrich spol. s.r.o, Prague, Czech Republic)/40 mL of DI mixture and kept for 24 h.

Immediately after TiO_2_ treatment, the membranes were taken and washed several times using DI water via the immersion method and one-minute cleaning with the ultrasonic cleaner to remove excessive TiO_2_ on the surface of the membrane.

**Figure 1:**
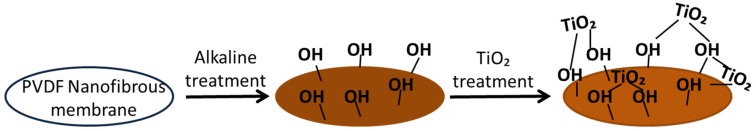
Surface modification of the polyvinylidene fluoride (PVDF) membrane.

Hydroxyl groups play an important role in the reaction chemistry of metal-oxide surfaces such as TiO_2_. The reaction of the alkaline solution with PVDF and the interaction between the dehydrofluorinated PVDF and TiO_2_ are shown in Figure 1.

The interaction between dehydrofluorinated PVDF and TiO_2_ was studied in the literature [4,19,20].

### 2.3. Characterization

Surface morphology of the membranes was characterized using scanning electron microscopy (SEM, Vega 3SB, Brno, Czech Republic). The samples were mounted on a stub of metal with adhesive (double-sided adhesive), coated with 7 nm of gold, and then observed in the microscope under various magnifications at various places (accelerating voltage = 30 kV; beam intensity = 7). Fiber diameter was analyzed using the free online Image-J program. From each sample, at least 50 fibers were measured.

Average, maximum, and minimum pore sizes of the membranes were measured using a custom-made porometer and the principle of bubble point measurement, as explained in the literature [15]. The bubble point test is used to determine the size of the pores of the porous material. In the bubble point test, sufficient gas pressure is applied to overcome the capillary forces of the wetted membrane pores to determine largest pore size. In this method, it is necessary to control the pressure needed to pass a liquid through the tested porous material and for wetting the sample. The size of the average and minimum pores can also be determined by increasing the air pressure and measuring its flow through the sample. In these circumstances, it is necessary to compare the pressure curve of the wet sample with the pressure curve of the dry sample. The flow rate increases when the pressure increases in the dry sample. On the other hand, in the wet sample, at the beginning, there is no flow because all the pores are filled with the liquid. At a certain pressure, the gas empties the largest pore, and gas begins to flow through the wet sample. The intersection between the calculated half-dry and the wet sample gives the mean flow pore size. When all the pores are emptied, an intersection between the wet and dry curve will be observed. This means that the relationship between the applied pressure and the detected flow becomes linear, and the intersection of the wet and dry curve represents the detected minimum pore size. In this work, ethylene glycol (surface tension 47.3 mN∙m^−1^) was used to wet the samples. Both wet and dry measurement were taken to determine maximum, minimum, and mean pore size. At least three measurements were taken. 

Air permeability of the membranes was tested using an SDL ATLAS Air Permeability Tester (Rock Hill ATLAS Air Permeability Tester (at 200 Pa and 20 cm^2^, Rock Hill, SC, USA). The air permeability test was used for determination of the air permeability of the flat membrane. A specimen was clamped over the test head opening by pressing down the clamping arm, which started the vacuum pump. Measurements were performed by application of 200 Pa of air pressure per 20 cm^2^ of fabric surface. At least three measurements were taken at various places on the membrane. Results were expressed as L·m^−2^·s^−1^.

Bursting pressure of the membranes was tested, and the maximum delamination pressure was recorded using a custom-made device. The membrane (47 mm in diameter) was placed between two rings, and pressurized water was applied from one side until the nanofiber layer delaminated from the supporting layer. The hydrostatic pressure was measured using a pressure controller, which was placed in front of the membrane and connected to a computer. The hydrostatic pressure was increased gradually, and, as soon as the nanofiber layer burst, the pressure value on the screen decreased sharply. The maximum pressure value was recorded as the bursting/delamination strength of the membrane [15]. 

A Krüss Drop Shape Analyzer DS4 (Krüss GmbH, Hamburg, Germany) was used for the measurement of water contact angle using distilled water (surface tension 72.0 mN·m^−1^). Five measurements from each membrane were taken.

The hydroxyl groups on the PVDF nanofiber were observed using Fourier-transform infrared spectroscopy (FTIR, Nicolet iZ10 by Thermo Scientific, Prague, Czech Republic).

### 2.4. Emulsion Preparation

A 50 vol.%/50 vol.% oil/water emulsion was prepared. Water-soluble/oil-insoluble food colorant was used to detect permeate after separation. Nonionic Triton X-100 (Sigma-Aldrich spol. s.r.o, Prague, Czech Republic) was used as a surfactant for preparation of the emulsion. Generally, oil–water emulsions are prepared using nonionic surfactants [21]. The preparation method was as follows:

⟶ A few drops of pink color food colorant were mixed with 100 g of distilled water.
⟶ Then, 2 g of surfactant was added to the water and mixed with a magnetic stirrer for 10 min.
⟶ Next, 100 g of sunflower oil was added to the water/surfactant mixture.
⟶ Finally, the solution was mixed with a magnetic stirrer at 500 rpm for 24 h.

**Figure 2:**
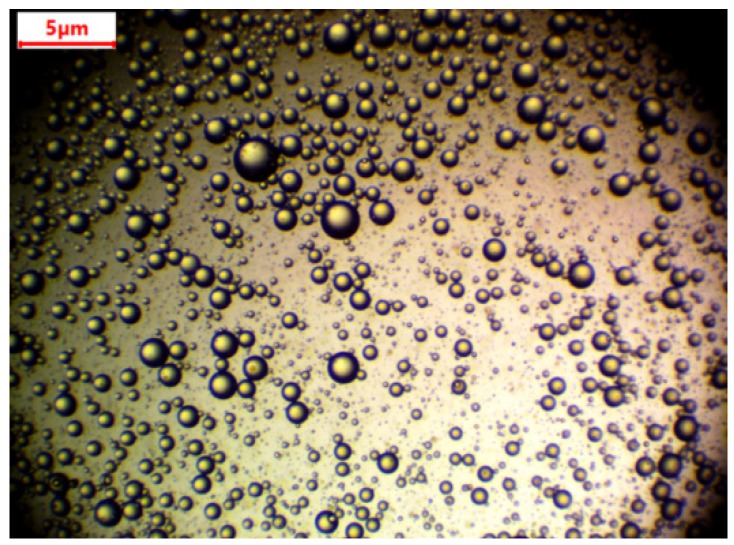
Droplets of oil under a microscope.

A digital microscope (Levenhuk Digital Microscope, Prague, Czech Republic) was used for the determination of oil droplet size (Figure 2). The emulsion was kept for one week without any stirring. Droplet size was then measured. There was no change in the size of the droplets. Average drop size was found to be 1.05 ± 0.34 µm.

### 2.5. Filtration Test

An Amicon (50 mL stirred cell, Millipore Corporation, Billerica, MA, USA) dead-end filtration unit was used for the separation test. The flux and the permeability of the samples were calculated according to Equations (1) and (2).

(1) $$F=\frac{G}{\mathrm{At}},$$

(2) $$P=\frac{F}{T},$$

where F is the flux (L·m^−2^·h^−1^), A is the area of the membrane (m^2^), G is the amount of permeate (L), t is the time of the filtration process, T is the transmembrane pressure, and P is the permeability of the membrane (L·m^−2^·h^−1^·bar^−1^).

Selectivity of the membranes was observed according to permeate color, and the microscope was used to detect any oil droplets in the permeate. The separation test for one membrane was done a number of times. In the first step, only 15 mL of distilled water was used as feed; in the second step, 15 mL of emulsion was used. This process was repeated at least three times to observe membrane fouling or self-cleaning.

## 3. Results and Discussion

### 3.1. Characterization of the Membranes

**Figure 3:**
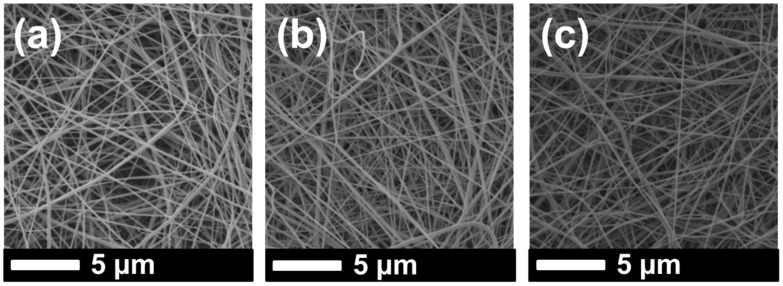
SEM images of (**a**) PVDF 1, (**b**) PVDF 2, and (**c**) PVDF 3 after lamination.

**Figure 4:**
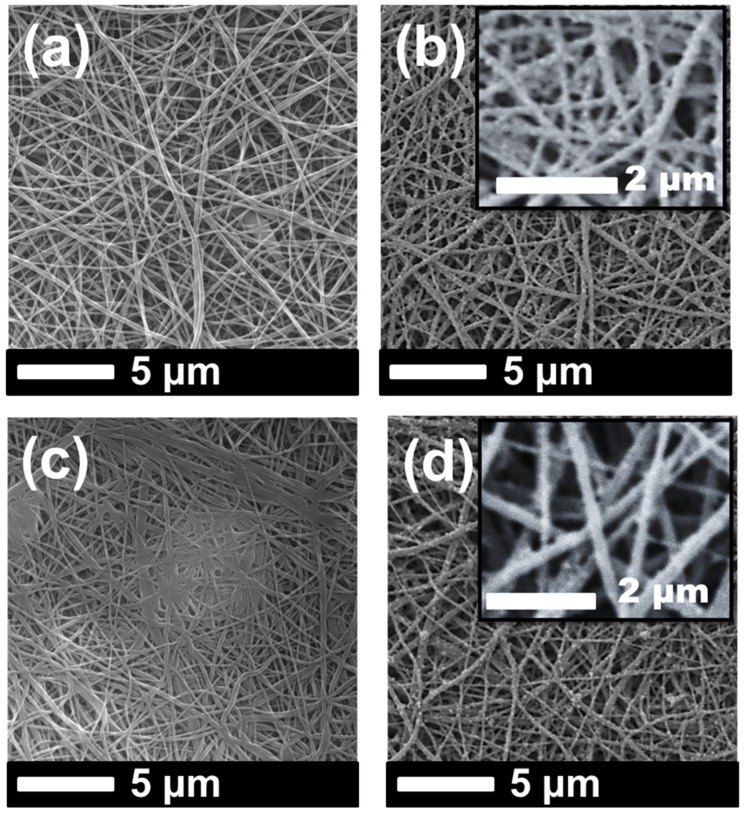
SEM images of (**a**) PVDF_N, (**b**) PVDF_NT, (**c**) PVDF_K, and (**d**) PVDF_KT.

SEM images of the samples were taken after lamination and the surface modification process (Figure 3 and Figure 4).

It was observed that the fiber diameter did not change with increasing nanofiber web density (Figure 3, Table 1). Since spinning conditions remained the same, only the backing paper speed was changed to get various nanofiber web densities, which did not influence fiber diameter. On the other hand, treatment of the PVDF nanofiber with an alkaline solution yielded a slight increase in fiber diameter because of swelling of the fibers (Figure 4a,c). Figure 4b,d show that TiO_2_ nanoparticles distributed very well on the surface of the nanofiber without any aggregation, showing that a regular dehydrofluorination took place. Hydrophilic OH groups on the membrane attached to the TiO_2_ nanoparticles. The fibers became thicker after the TiO_2_ nanoparticle attachment on the surface.

**Figure 5:**
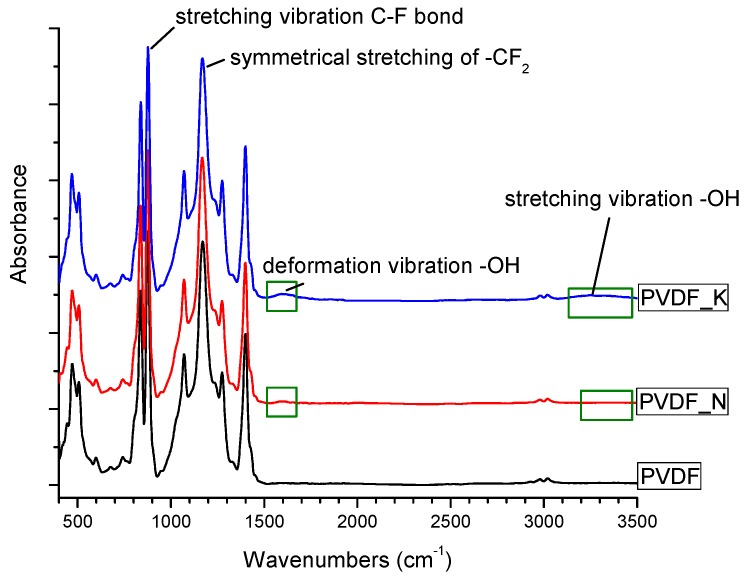
Fourier-transform infrared (FTIR) image of the PVDF membranes.

The FTIR spectra were collected in order to investigate the chemical structure of the PVDF nanofibrous webs. These are shown in Figure 5. The stretching bands at 1173 cm^−1^ and 876 cm^−1^ were attributed to the –CF_2_ and C–F groups of PVDF. The spectra confirmed the presence of –OH groups after surface modification, with absorption bands at 1600 cm^−1^ representing –OH group deformation vibrations. The very broad and less intense peak between 2500 cm^−1^ and 3500 cm^−1^ was due to O–H functionalities. It may be concluded that the bonded –OH groups played a major role in the hydrophilicity of the membranes.

Pore size of the samples before modification was measured and is shown in Table 2. Membrane pore size for the modified samples was not measured. Once the samples were modified, they were kept wet in distilled water as recommended. It was found that drying of the modified and wetted membrane could cause possible membrane cracks and damage. Unfortunately, pore size measurement was done only for the dry samples. Increased nanofiber web density caused a decrease in the average pore size due to the compact structure. The role of pore size in the separation process was significant. Tight pore size increased the selectivity of the membranes.

**Figure 6:**
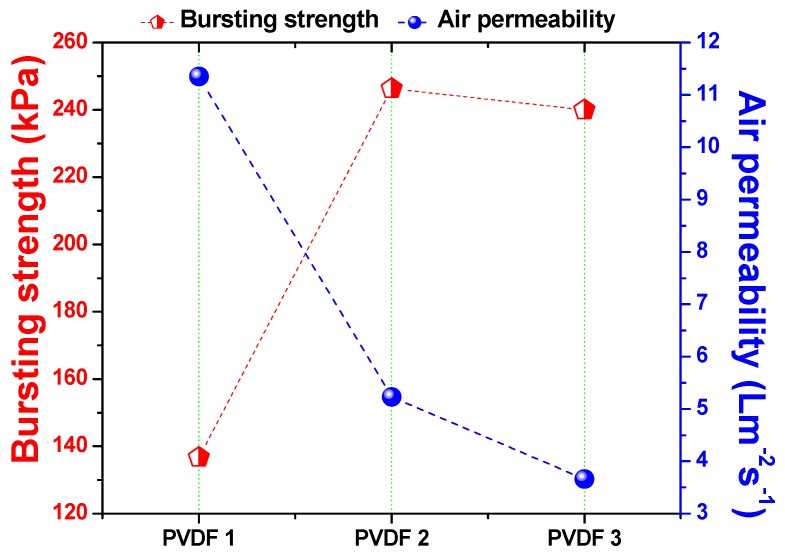
Bursting pressure and air permeability of the membrane according to nanofiber web density.

Air permeability of the membranes was measured after the lamination process. The main aim of this step was to observe whether the adhesive blocked the pore size of the nanofibers during lamination. The bursting pressure test is another method of determining the quality of the lamination process. Using this test, the strength of the lamination was measured. Figure 6 shows the relationship among density of the nanofiber layer, air permeability, and bursting pressure.

There was an inverse proportional relationship between the density of the nanofiber web and air permeability. Lower density meant fewer fiber bundles on the web, which resulted in a more open structure. On the contrary, higher density meant more fiber bundles, which resulted in a compact structure. Bursting pressure results indicated that, at the lowest density (1 g/m^2^), adhesion of the nanofibers to the support was not as strong as that at the higher nanofiber web density. The reason could have been the low mechanical strength and high abrasion resistance of the low-density nanofiber fiber web. As soon as the fiber density increased from 1 to 2 g/m^2^, bursting pressure improved by 80%.

Further improvements did not change fiber density significantly. Based on previous work [8,13], the minimum required bursting pressures for the PVDF and polyacrylonitrile (PAN) nanofiber membranes were determined to be 175 kPa and 195 kPa, respectively. When density exceeded 1 g/m^2^, nanofibers showed a bursting pressure >175 kPa. It can be suggested that the minimum required nanofiber web density is 2 g/m^2^ for the preparation of membranes. Since the membrane with 3 g/m^2^ showed excellent bursting pressure, and the possibility of modification of more fibers on the surface, this membrane was selected as the candidate for surface modification.

Water contact angle (CA) of the membranes was measured. Membrane behavior under emulsion changed due to additives such as surfactant. The CA of the membranes was measured after oil separation. Results are given in Table 3.

The CA of the neat membranes without any modification had a contact angle <90°, which can be considered “hydrophilic”. Typically, PVDF nanofibers have a hydrophobic nature. After lamination, the surface structure of the PVDF membranes most likely changed. Moreover, the adhesive web between the nanofiber and the supporting layer played a significant role. The adhesive web partly covered the surface of the nanofibers, which may have exhibited hydrophilic characteristics.

Results of the CA showed that increasing the density of the nanofiber web decreased the wettability of the samples. There was a proportional relationship between nanofiber web density and CA.

Research results indicated that increasing the hydrophilicity of the membrane prevented membrane fouling and improved membrane permeability [4,16,22].

### 3.2. Separation Test

**Figure 7:**
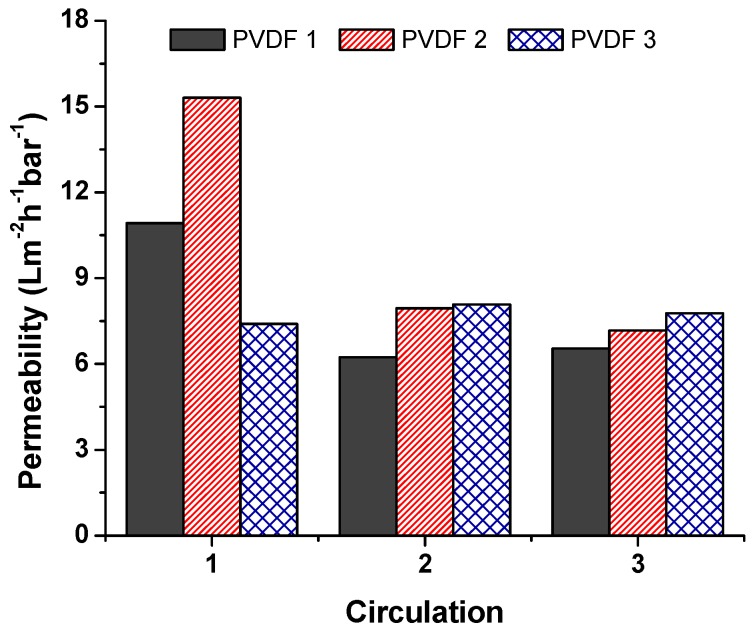
Permeability of the unmodified membranes.

A separation test was run using dead-end cell separation, and the permeability of the unmodified membranes was calculated according to Equation (2). Results are given in Figure 7, which compares unmodified membranes at various densities. Each membrane was used and circulated three times to measure fouling. Between each circulation, distilled water was filtrated. Results indicated that, at the beginning, PVDF 2 showed enormous permeability compared to the others. The lowest permeability was achieved using PVDF 3 at the first circulation. However, membranes PVDF 1 and PVDF 2 showed a sharp decrease in permeability with the second circulation; the reason for this was membrane fouling. The membrane with the highest nanofiber density (PVDF 3) showed a stable permeability after three circulations; this could have been due to the high specific surface area of PVDF 3. A higher density meant more nanofiber web was on the structure, resulting in a bigger surface area in total. On the contrary, Hobbs et al. [23] found that there is a proportional relationship between flux decline ratio and membrane surface area. A higher surface area showed a higher flux decline ratio.

**Figure 8:**
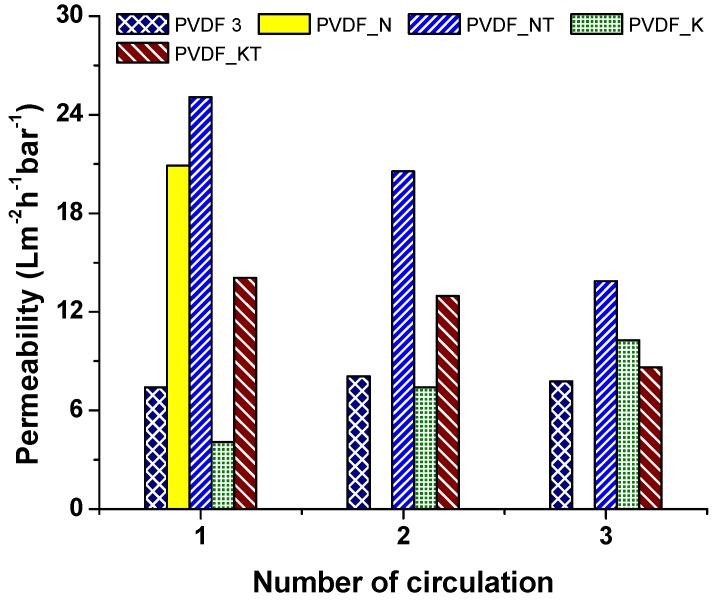
Permeability of the modified membranes.

Figure 8 shows the results of the modified membranes. Surface modification helped improve membrane surface cleaning. For long-term application, surface-modified membranes should be an excellent candidate for use in the separation process. Life span and performance of the membranes were improved using surface modification. Moreover, the added TiO_2_ acted as an antibacterial on the membranes. The TiO_2_ nanoparticles were activated under ultraviolet (UV) light during separation, which might have enhanced the performance, self-cleaning, and antibacterial properties of the membranes [24]. Montazer et al. [25] modified the surface of polyester/wool fabric using TiO_2_ nanoparticles. Water adsorption increased, while the time of water droplet adsorption decreased.

Moreover, the antibacterial efficacy of the material against *Escherichia coli* was found to be 100%. In another study, it was reported that a polydimethylsiloxane (PDMS)/TiO_2_ composite provided excellent photocatalytic properties and developed self-cleaning properties [26]. Xu et al. [27] prepared TiO_2_–high-density polyethylene (HDPE) nanocomposite surfaces that exhibited superhydrophobicity. Exposure to UV light caused the surface of the composite to become hydrophilic. As a result, wettability and self-cleaning properties of the nanocomposite increased. More examples appeared in the literature. The effectiveness of TiO_2_ nanoparticles on wettability and self-cleaning properties is indisputable.

**Figure 9:**
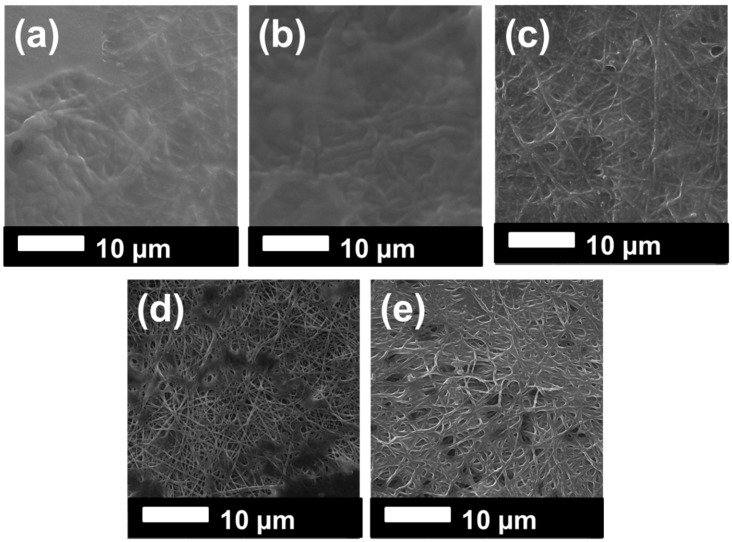
SEM images of the samples after oil separation: (**a**) PVDF 3, (**b**) PVDF_N, (**c**) PVDF_NT, (**d**) PVDF_K, (**e**) PVDF_KT.

The surface of the membranes after oil separation was detected using SEM images, as shown in Figure 9. We could understand from the SEM images that membranes with high permeability and surface cleaning attracted less oil. For instance, the permeability of the PVDF 3 decreased consistently in each circulation. PVDF_N was blocked after the first circulation. These two membranes showed, in the SEM images (Figure 9a,b), that an oily film covered the surface of the membranes. For the membranes PVDF_N and PVDF_NT, there was less oil contamination on the membrane surface. Based on this result, it can be concluded that surface modification improved membrane permeability and self-cleaning performance.

Permeate solution was collected and checked under the microscope to detect any oil droplets. Moreover, coloring the water helped to detect oil content in the mixture. Unmodified membranes showed hydrophobic/oleophilic characteristics, while modified membranes were hydrophilic/oleophobic.

## 4. Conclusions

PVDF is one of the most frequently used polymers in the membrane filtration market due to its outstanding properties, such as chemical resistance, thermal stability, and high mechanical strength. Despite the superior properties of PVDF membranes, there is still plenty of room for improvement in membrane performance and life span. In this work, PVDF nanofibrous hybrid membranes were prepared for the separation of the oil–water emulsions. A two-step surface modification took place using alkaline solution and TiO_2_ nanoparticle grafting. Water permeability of the membranes increased due to –OH groups and hydrophilic TiO_2_ nanoparticles (NPs) on the membrane surface.

Moreover, membranes showed self-cleaning properties after the modification process. Photocatalytic activity can be improved through UV induction on the TiO_2_-modified membranes. We believe that this method can be used in the separation of emulsified oil/water. Based on the result, it can be concluded that nanofiber webs are good candidates for water domain filtration applications.

## Figures and Tables

**Table 1:** Abbreviations of the samples. PVDF—polyvinylidene fluoride.

Polymer	Density (g/m^2^)	Modification	Abbreviation	Fiber Diameter (nm)
PVDF	1	-	PVDF 1	148.9 ± 23.1
	2	-	PVDF 2	148.5 ± 45.6
	3	-	PVDF 3	134.2 ± 37.2
	3	NaOH	PVDF_N	164.9 ± 40.3
	3	NaOH + TiO_2_	PVDF_NT	248.2 ± 47.8
	3	KOH	PVDF_K	174.9 ± 57.6
	3	KOH + TiO_2_	PVDF_KT	197.0 ± 54.7

**Table 2:** Maximum and average pore size of the membranes.

Sample	Maximum Pore Size (µm)	Average Pore Size (µm)
PVDF 1	4.54 ± 0.14	2.50 ± 0.29
PVDF 2	4.20 ± 0.00	1.15 ± 0.08
PVDF 3	4.23 ± 0.05	0.72 ± 0.04

**Table 3:** Water contact angle (CA) of the membranes before and after separation.

Sample	CA before Separation (°)	CA after Separation (°)	Image (Before Separation)
PVDF 1	71.23 ± 1.31	62.40 ± 2.17	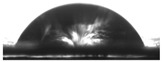
PVDF 2	80.83 ± 1.53	47.46 ± 1.93	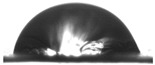
PVDF 3	89.40 ± 4.67	35.32 ± 8.71	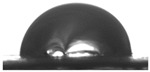
PVDF_N	0	0	-
PVDF_NT	39.43 ± 3.01	0	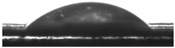
PVDF_K	0	0	-
PVDF_KT	0	0	-
